# Ethiopic maternal care data mining: discovering the factors that affect postnatal care visit in Ethiopia

**DOI:** 10.1186/s13755-016-0017-2

**Published:** 2016-05-23

**Authors:** Geletaw Sahle

**Affiliations:** School of Computing, Jimma Institute of Technology, Jimma University, Jimma, Ethiopia

**Keywords:** Data mining, Maternal health, J48, JRip, Postnatal care

## Abstract

**Background:**

Improving maternal health and reducing maternal mortality rate are key concerns. One of the eight millennium development goals adopted at the millennium summit, was to improve maternal health in Ethiopia. This leads towards discovering the factors that hinder postnatal care visit in Ethiopia.

**Methods:**

In this research, knowledge discovery from data (KDD) was applied to identify the factors that hinder postnatal care visits in Ethiopia. Decision tree (using J48 algorithm) and rule induction (using JRip algorithm) techniques were applied on 6558 records of Ethiopian demographic and health survey data. To construct essential target dataset attributes exploratory data analysis with frequency diagram is performed, missing value was filled and noisy value was corrected. Also the data are preprocessed using business and data understanding with detail statistical summary.

**Result:**

J48 (93.97 % accuracy) and JRip (93.93 % accuracy) identifies places of delivery, assistance of health delivery professional, prenatal care health professional and age are the determinant factors. However, residence places also taken into consideration.

**Conclusions:**

In this study, encouraging results were obtained by employing both decision tree and rule induction techniques. The rules generated by J48 and JRip algorithms are much understandable to explain the outcome easily. Thus, the result obtained highly supportive to construct, evaluate and update advertising and promotional maternal health policies. It is better to create a generic model with more coverage in terms of economic, demographic, social and genetic factors so as to integrate the result with knowledge based system.

## Background

Maternal health care is a concept that encompasses family planning, preconception, prenatal, and postnatal care. More specifically it refers to the health of women during pregnancy, child birth and postpartum period [1]. Ethiopia has been working to reduce maternal and child mortality rate for a decade. Now a days government and non-government organizations in collaboration with Ethiopian Federal Ministry of Health (EFMH) provide proper education, health promotion, screening and intervention which helps to reduce the risk factors that might occur during pregnancy. Even though the efforts are remarkable due to lack of proper intervention complications of pregnancy and child birth are the leading causes of death in developing countries [2].

World Health Organization (WHO) global report shows that an estimated 289,000 women died during pregnancy and childbirth. In sub-Saharan Africa a woman’s life time risk of dying during pregnancy and childbirth is 1 in 3700, the risk of maternal death is very high at 1 in 38 [3]. United Nations Children’s Fund (UNICEF) and United States Agency for International Development (USAID) noted many children left motherless in each year, maternal mortality rate continues at an unacceptably high rate and girls die each year due to pregnancy-related complications. According to UNICEF “on average 8 million babies die before or during delivery or in the first one week of life”. The direct cause of maternal deaths are hemorrhage, infection, obstructed labour, hypertensive disorders in pregnancy, and complications of unsafe or unsanitary abortions [4]. Poor maternal health, nutrition and quality of care at delivery are the main cause of disease for children below the age of five and it shares twenty percent of the burden. USAID [5] strengthen the report of WHO and UNICEF by stating more than 500,000 Ethiopian women and girls suffer from disabilities caused by complications during pregnancy and childbirth each year.

Central Statistical Agency indicates that “*15 percent of pregnancies and childbirths need emergency obstetric care because of complications that are difficult to predict*” [6]. The report boldly noted that due to the limitation of mining tools the pattern of maternal health data could not become an opportunity to mitigate the burden of maternal deaths and disabilities. Exploring these pattern (hidden knowledge) helps to guide the advocacy effort and research at national level.

Ethiopian Federal Ministry of Health emphasizes women who begin prenatal care early in their pregnancies have better birth outcomes than women who receive little or no care during their pregnancies [7]. This leads towards prenatal care (a care that a women receive and provide for themselves throughout their pregnancy) and postnatal care (include recovery from childbirth, concerns about newborn care, nutrition, breastfeeding, and family planning).

Therefore; the aim of this study was to investigate the factors that affect postnatal care visit using data mining which can support primary health care providers, policy makers, and planners to identify the major determinants of maternal health causes, prevent and control maternal mortality rate. Since health program largely relies on timely and accurate information, identifying health-related problems are important in planning of healthcare interventions. More specifically this research tries to answer which group of mothers are likely to attend postnatal care after giving birth, which group of mothers are at greater risk of mortality and morbidity, which attributes are more important to postnatal service care and which segment of the mother’s best fit with the current service capacities of postnatal care.

## Related works

Reducing maternal and child death in developing countries face numerous challenges due to lack of proper education, health promotion, screening and interventions. Investigating the factors that affect postnatal care visit will have a great contribution towards eradicating the maternal and child death.

Maimon and Kandel [8] applied knowledge discovery on a dataset of 33,134 mortality rate records extracted from the Israeli Ministry of Health mortality database. Information-theoretic to data mining approach used to identify the leading causes of death and the association of various factors with certain diseases. Prather et al. [9] took two-year sample dataset (1993–1994) from comprehensive longitudinal medical record system at Duke University to identify the factors that improve the quality and cost effectiveness of prenatal care. Chawani et al. [10] attempts to balance the work practices and protocols in the development of an EMR system for antenatal care in Malawi. They tried to answer how ICTs could be used to improve the maternal healthcare services and discovered clients in healthcare system has crucial for the development of effective EMR system. Darteh et al. [11] address the association between women’s economic and socio-demographic characteristics and their decision making on engaging in sexual intercourse and use of condom using multivariate logistic regression. D’Souza et al. [12] qualitative study using purposive sampling discover the factors that affect women’s reproductive health among married in mining communities in India. Markos et al. [13] build a predictive model using PART pruned rule induction for checking the nutrition status of under 5 years children.

The above effort tried to explore the leading cause of maternal mortality rates, factors that affect the quality and cost effectiveness of prenatal care, the association between maternal care services and socio-economic effects on maternal care including reproductive health and nutritional status. However, understanding the case of high child and maternal mortality as well as morbidity in developing countries like Ethiopia need further investigation. If we can determine/mine the hierarchical and predictive importance of different risk factors and their patterns using data mining majority of maternal deaths and disabilities are preventable [14]. Identifying those factors used as to take preventive actions, improve postnatal care and their survival. In this research, classification data mining techniques are applied for extracting hidden knowledge and important patterns from large volume of data, based on which predictive modeling is constructed that can help in decision making process [15, 16]. Hence, the purpose of this research is to explore the factors that hinder/affect postnatal care visit.

## Methods

### Data exploration and preprocessing

Knowledge discovery from data was adopted to investigate the factors that hinder postnatal care visits in Ethiopia among 6558 records. We used secondary data from Ethiopia Demographic and Health Survey (EDHS) 2011 dataset. The dataset was conducted under the support of ministry of health and central statistical agency, in every 5 years of interval. The dataset covers family planning, fertility levels and determinants, fertility preferences, infant, child, adult and maternal mortality, maternal and child health, nutrition, malaria, and women’s empowerment. The primary objective was to provide up-to-date information for policy makers, planners, researchers and programme managers, which would allow guidance in the planning, implementing, monitoring and evaluating of population and health programmes in the country [14].

To understand the problem domain we used observation, interviewing with domain experts and data managers, reviewing documents, reports and literatures. This helps us to maintain the data quality, select and integrate decisive attributes. To prepare the data for analysis and build suitable predictive model, preprocessing tasks such as data cleaning (for filling missing values, handling noisy and outlier values), data integration and transformation tasks are performed.

**Fig. 1:**
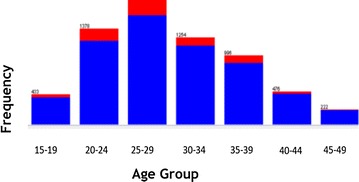
Summary of women distribution by age

**Fig. 2:**
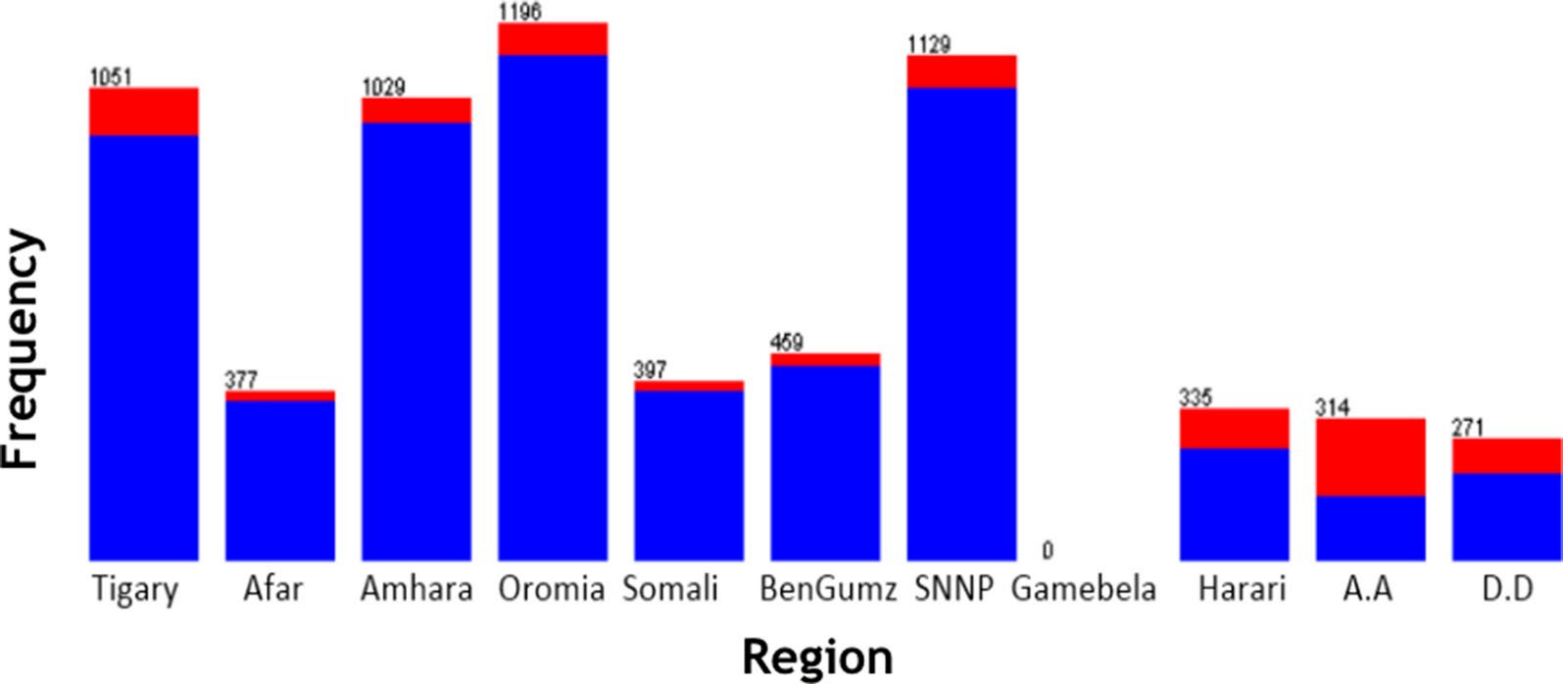
Summary of women distribution by region

**Fig. 3:**
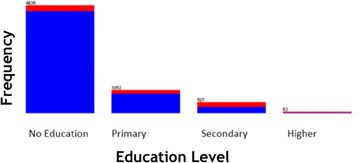
Summary of women distribution by education level

**Fig. 4:**
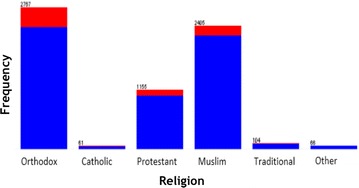
Summary of women distribution by religion

WEKA exploratory data analysis helps to get the familiarity of the data and prepare them for mining [17]. Exploratory data analysis with frequency diagram was performed to detect attributes with missing value and wrong entry. Data cleaning and pre-processing tasks like decoding of inconsistent data encoding and handling missing value has performed. The data mining task is to uncover the factors that hinder postnatal care visit using J48 and JRip classification algorithms. The description of selected attribute together with exploratory data analysis has mentioned in Figs. 1, 2, 3 and 4. The blue color in the frequency diagram indicates those who attend postnatal care visit and the red indicates those who did not attend postnatal care.

### Age

The age of mothers is classified by 5 year age groups. This attribute is categorized into seven groups: 15–19, 20–24, 25–29, 30–34, 35–39, 40–44 and 45–49 as shown in the Fig. 1.

### Region

The region attribute indicates the location where the mothers residues. This attribute contains a total of 11 administrative region as shown in Fig. 2.

### Education

This attribute reveals the education level of a mother. Mother’s education is indirectly related to a child’s health. Mother’s education is nominal attribute that contains four distinct values (no education, primary, secondary, and higher) as shown in Fig. 3.

### Religion

This attribute indicate the religion of the mother. Mother’s education is nominal attribute that contains six distinct values (Orthodox, Catholic, Protestant, Muslim, Traditional and other) as shown in Fig. 4.

In all the maternal health dataset contain nine main and sub sections. On average each sections have six attributes. Since region, residence, age, religion and education are similar for all section we merge and aggregate them together to generate the final 14 attributes. Summary of the datasets compiled for building predictive model with their possible nominal values and description are depicted in Table 1 below.

**Table 1:** Summary of attributes and their values

No	Attributes	Description summary of attributes
1	Region	The location where the mother get delivery and maternal care. It is nominal value includes all the regions in Ethiopia such as Tigray, Afar, Amahra, Oromiya, Somali, BenGumz, SNNP, Gambela, Harari, Addis Ababa and Dire Dawa
2	Residence	The place where the mother live either rural or urban. It contain nominal value urban and rural
3	Age	The age of the mother. It contain nominal value with the age group of 15–19,20–24,25–29,30–34,35–40,40–44 and 45–49
4	Religion	Indicates the religion of the mother belongs with nominal value such as orthodox, catholic, protestant, muslim, Traditional or other
5	Education	The education status of the mother categorized into no education, primary, secondary and higher
6	Tetanus injection	Tetanus injection before birth with a nominal value of no injection, received and do not know
7	PProfessional	Prenatal health professional with a nominal value of yes or not
8	Pother	Prenatal other professional like friends, family with a nominal value of yes or no
9	AhealthPDS	Assistance health professional during delivery Service with a nominal value of yes or not
10	Auntrain DS	Assistance untrained delivery service attendant with a nominal value of yes or not
11	NoneDS	Indicates no assistance during delivery with a nominal value of yes or not
12	DeliveryPlace	The place where the mother born their child with a nominal value of yes or not
13	Dlbycaesariansction	The mother who deliver their child by caesarian Section with a nominal value of yes or not
14	PostnatalCare	Maternal care taken after giving birth with a nominal value of yes or not

### Experimental setup

For experimental setup the data is partitioned into 10-fold cross validation. To identify the factors that hinder postnatal care visit J48 decision tree algorithm and rule induction (using JRip algorithm) experimented. J48 algorithm supports both numeric and nominal predicators and nominal class attribute values to generate output in tree and rule set forms [15]. Each rule describes a specific context associated with a class and easier to understand. In all, the output shows that the hierarchy of the determinant factors that hinder postnatal care visit. Rule induction using JRip has both the ability to produce accurate and readable rules [16]. The generated rules are in plain text form and simple to understand.

In the first part of the analysis all the 14 attributes were used to build model. We used the k-fold (k = 10) cross validation test options. Cross validation partition the dataset for training and testing. The testing dataset is removed before training begins. Once the training is done, the dataset that was removed can be used to test the performance of the learned model. By doing so the partition and the experiment could be more reliable. In this test option the accuracy estimate is the overall number of correct classifications from the k iteration divided by the total number of samples, which is k. After deciding the values of the parameters the algorithm was run to start building the model.

To build the model we used 14 attributes and interesting rules extracted from J48 tree and JRip algorithms. Experimental evaluation of models was taken place based on performance/accuracy of models and confusion matrix, informal discussion with the domain expert and based on the soundness of the rules generated. A total of 22 interesting rules generated (i.e. 15 in J48 decision tree algorithm and 7 in JRip rule induction algorithm) with an accuracy 93.9768 and 93.9311 % respectively. Out of 6558 record datasets J48 algorithm were classified 6163 records correctly and build a model with an accuracy of 93.9768 and 6.0232 % incorrect classified instance. JRip built a model with an accuracy of 93.9311 %.

**Fig. 5:**
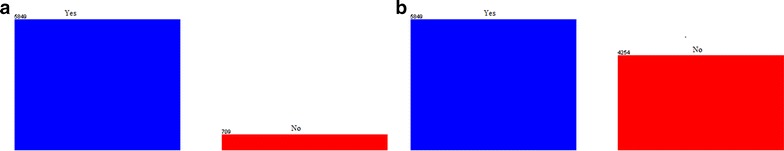
**a** and **b** postnatal care visit class

The model need further observation and evaluation since it is constructed from imbalanced dataset. As shown in Fig. 5a the dataset is imbalanced. We applied SMOTE (Synthetic Minority Oversampling Technique) to avoid the influence of the majority class (postnatal care visit) and to resolve the issue of class imbalance problem [18]. SMOTE is an over-sampling approach in which the minority class (not visiting postnatal care visit) is over-sampled by taking each minority class (not visiting postnatal care) sample and introducing synthetic examples along the line segments joining any/all of the k minority class nearest neighbors. The result of the dataset after SMOTE depicts in Fig. 5b.

**Fig. 6:**
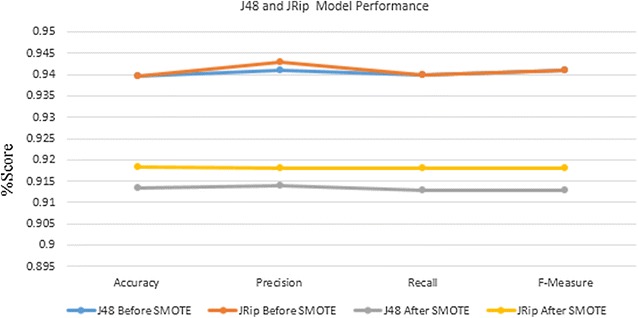
J48 and JRip model performance result

Figure 6 depicted comparison of the performance of J48 and JRip model before and after SMOTE using accuracy, precise, F-Measure and recall. J48 algorithm performs 93.97 % accuracy, 94.1 % precision, 94 % recall and 94.1 % F-Measure before SMOTE applied and 91.34 % accuracy, 91.4 % precision, 91.3 % recall and 91.3 % F-Measure using SMOTE. JRip performs 93.93 % accuracy, 94.3 % precision, 94 % recall and 94.1 F-Measure before SMOTE applied and 91.83 % accuracy, 91.8 % precision, 91.8 % recall and 91.8 F-Measure using SMOTE.

In all the result from SMOTE also confirmed the initial experiment and noted that assistance health professional during delivery, age, region, residence, delivery place, tetanus injection and prenatal health professional has great impact to promote postnatal care visit.

## Experimental results and discussions

Summary of experimental result for discovering the factor that hinder postnatal care visit using decision tree (using J48 algorithm) and rule induction (using JRip algorithm) are presented in “Rules generated from J48 algorithm” and “Rules generated from JRip algorithm” sections below.

### Rules generated from j48 algorithm

**Fig. 7:**
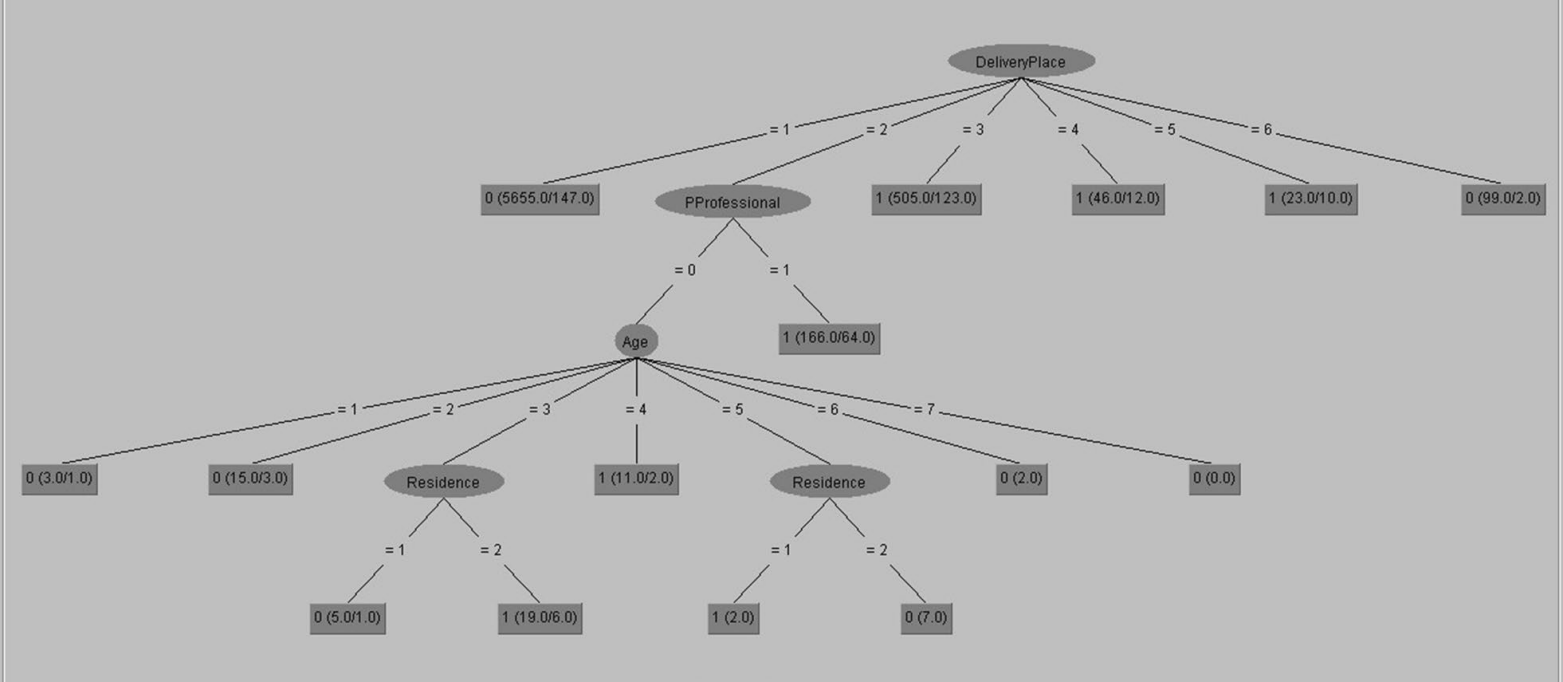
J48 output

J48 hierarchy features used to identify the most significant variable that used to discriminate the records (located at the top as depicted in Fig. 7). Each rule is taken by reading the J48 pruned tree following the path from the root node to each leaves that contains the dependent class values as summarized in Table 2.

**Table 2:** Interesting rule generated from J48 algorithm

Rule	Result (output)
1	If place of delivery is home they will not attend postnatal care service
2	If place of delivery is public health centers and prenatal care not conducted by health professional and the age group is 15–19 then they will not attend postnatal care service
3	If place of delivery is public health centers and prenatal care not conducted by health professional and the age group is 20–24 then they will not attend postnatal care service
4	If place of delivery is public health centers and prenatal care not conducted by health professional and the age group is 25–29 and types of place of residence is rural then they attend postnatal care service
5	If place of delivery is public health centers and prenatal care not conducted by health professional and the age group is 25–29 and types of place of residence is urban then they will not attend postnatal care service
6	If place of delivery is public health centers and prenatal care not conducted by health professional and the age group is 30–34 then they attend postnatal care service
7	If place of delivery is public health centers and prenatal care not conducted by health professional and the age group is 35–39 and types of place of residence is rural then they will not attend postnatal care service
8	If place of delivery is public health centers and prenatal care not conducted by health professional and the age group is 35–39 and types of place of residence is urban then they attend postnatal care service
9	If place of delivery is public health centers and prenatal care not conducted by health professional and the age group is 40–44 then they will not attend postnatal care service
10	If place of delivery is public health centers and prenatal care not conducted by health professional and the age group is 45–49 then they will not attend postnatal care service
11	If place of delivery is public health centers and prenatal care are conducted by health professional they attend postnatal care service
12	If place of delivery is government hospital they attend postnatal care service
13	If place of delivery is private health sector they attend postnatal care service
14	If place of delivery is NGO health facility they attend postnatal care service
15	If place of delivery is others they attend postnatal care service

Summary of experimental results executed using J48 algorithm are presented in Table 2 below.

### Rules generated from JRip algorithm

Summary of experimental results executed using JRip algorithm are presented in Table 3 below. The number in the bracket stands for coverage/errors in the training data, which follows the standard convention of tree/rule induction. e.g. (AhealthPDS = 1) and (DeliveryPlace = 4) ≥ PostnatalCare = 1 (46.0/12.0) means that the rule “(AhealthPDS = 1) and (DeliveryPlace = 4) ≥ PostnatalCare = 1 cover instances with total weight of 46.0, out of which there are instances with weight of 12.0 misclassified. Normally weight 1 means one instance.

**Table 3:** Interesting rule generated from JRip algorithm

Rule	Result (output)	Interpretation (the mother will attend postnatal care)
1	(A health PDS = 1) and (Delivery place = 3) ≥ Postnatal care = 1 (505.0/123.0)	If she is assisted by health professional during delivery Service in government hospital
2	(A health PDS = 1) and (Delivery place = 2) ≥ Postnatal care = 1 (225.0/96.0)	If she is assisted by health professional during delivery Service in public health center
3	(A health PDS = 1) and (Delivery place = 4) ≥ Postnatal care = 1 (46.0/12.0)	If she is assisted by health professional during delivery Service in NGO health facilities
4	(Residence = 1) and (A health PDS = 1) and (Delivery place = 5) ≥ Postnatal care = 1 (6.0/2.0)	If she is assisted by health professional during delivery Service in private health center and lives in urban area
5	(A health PDS = 1) and (Delivery place = 5) and (Region = 7) ≥ Postnatal care = 1 (4.0/0.0)	If she is assisted by health professional during delivery Service in private health center in SNNP region
6	Postnatal care = 0 (5772.0/156.0)	The rest did not attend postnatal care visit

### Analysis and discussion

J48 algorithm identified the most determinant factors to predict maternal healthcare visit after birth with 93.97 % accuracy as presented in Table 2. Delivery place, prenatal health professional and age are found to be the determinant factors for attending postnatal care. Rules derived from 1, 2, 3, 5, 7, 9, 10 and 15 indicates that those mother who deliver at home and public health center will not attend postnatal care visit. Specially, those mother who deliver at public health centers (government hospital, public health center, private health sector and NGO health facility) and did not attend prenatal care by health professional with age group of 15–19, 20–24, 40–44 and 45–49, place of residence urban with age group of 25–29 (needs further investigation) and place of residence rural with age group of 35–39.

Rule 4 shows that if a mother deliver at public health centers and did not attend prenatal care by health professional with age group of 25–29 and residence in rural then they will attend postnatal care service. It creates interesting rule and needs further investigation. As mentioned in rule 6 and 8, those mother who gave birth at public health centers and did not attend prenatal care by health professional with age group of 30–34 and 35–39 and residence in urban area will attend postnatal care automatically.

Rule 11, 12, 13 and 14 shows that most of the time, those mother who gave birth at health center and attends prenatal care by health professional will attend postnatal care automatically i.e. place of delivery at public health centers and attend prenatal care by health professional, government hospital, private health sector and NGO health facility.

Table 3 presents clear and understandable rule generated using JRip. JRip with 93.93 % accuracy identifies assistance health professionals during delivery and delivery place are the most determinant factors. Delivery place such as government hospital, public health center, private health sector and NGO health facility are a driving force for postnatal health care visits. JRip result indicates the delivery place must be accessible for mothers and the country tries to increase health professionals as much as possible.

In general interesting rule were generated to indicate whether a mother attends postnatal care or not with respect to different attributes during their maternal postnatal care service, from both rules we observe postnatal care visits is not a trend throughout the country. The result of this research shows that delivery place, prenatal health professional and age as well as residence are important variables to predict postnatal health care services.

In all, both techniques indicates that delivery place must be accessible for all mothers and assist prenatal and postnatal care using health professionals as much as possible. In general the result from this study were encouraging to support ministry of health and other responsible bodies. However, in order to improve postnatal care and their complications of mother health after delivery a model handling datasets of economic and genetic factors required. The Federal Ministry of Health focus their attention on these factors by constructing rules and policies to eradicate and control maternal mortality and morbidity.

## Conclusion and future work

The study tried to identify the factors that hinder postnatal care visits in Ethiopia. Place of delivery, assistance health delivery professional, prenatal care health professional and age are the determinant factors that affect postnatal care visit. Residence place also take into consideration to reduce maternal mortality and morbidity as well as disability due to ignorance of postnatal care visits.

In this study, encouraging results were obtained by employing both decision tree and rule induction techniques. The rule generated by J48 and JRip algorithms are much understandable to explain the prediction outcome easily. Thus, the result obtained highly supportive to construct, evaluate and update advertising and promotional maternal health policies. To design an optimal strategy a generic model with more coverage in terms of economic, demographic, social and genetic factors so as to integrate the result of data mining with knowledge based system.
